# The effect of an internet option and single-sided printing format to increase the response rate to a population-based study: a randomized controlled trial

**DOI:** 10.1186/1471-2288-14-104

**Published:** 2014-09-09

**Authors:** Elisa Flüß, Christine M Bond, Gareth T Jones, Gary J Macfarlane

**Affiliations:** 1 Epidemiology Group, University of Aberdeen, Polwarth Building, Foresterhill, Aberdeen AB25 2ZD, UK; 2 Centre for Academic Primary Care, University of Aberdeen, Polwarth Building, Foresterhill, Aberdeen AB25 2ZD, UK

**Keywords:** Response rate, Randomized controlled trial, Postal questionnaires, Health surveys, Data collection, Internet

## Abstract

**Background:**

Paper questionnaires are a common means to collect self-reported information in population-based epidemiological studies. Over the past decades, the response rates to epidemiological studies have been decreasing which can affect the selection process of eligible subjects and lead to non-response bias. Hence, research into strategies to increase questionnaire response rates is crucial. The aim of this study was therefore to explore the effectiveness of single-sided questionnaires and an internet option for response in increasing response rates to a population-based study.

**Methods:**

A 2×2 factorial experiment was embedded within a large population-based study of pain and pain management. Persons in the study sample were 4600 residents in Grampian (north of Scotland) aged 25 years and over who were randomly selected from health board records. Sampled persons were randomly assigned to either receive a single-sided or double-sided questionnaire with or without an internet option to respond. The study questionnaire was distributed via post.

**Results:**

The overall study response rate was 36.3%. When compared to the reference group that received no intervention (response rate = 35.5%), the response rate changed only marginally when single-sided questionnaires were distributed (35.8%) or when an option to reply via the internet was provided (34.3%). A somewhat higher increase in response rates was achieved when both strategies were employed (39.6%). Overall, no significant effect on response rate was determined for each strategy or their interaction.

**Conclusions:**

Evidence from this study suggests that neither single-sided questionnaires nor the option to reply via the internet resulted in a significant increase in response rates to population-based studies.

## Background

Questionnaires to collect self-reported information are one of the key elements in population-based studies. A common means to conduct these studies is the use of postally distributed questionnaires which are a convenient way to reach a large number of people in a short period of time [1]. While population studies are less prone to selection bias, low study response rates and selective participation can result in non-response bias which can threaten a study’s validity [2].

It has been widely observed that response rates to epidemiological studies have been decreasing over the past decades [3,4]. A retrospective review to investigate the change in study participation over time, reported that between 1970 and 2003 the response rates to cohort and cross-sectional studies decreased by −0.54% (95 CI: −1.33, 0.24) and −0.67% (95% CI: −1.91, 0.56) per year, respectively [4]. According to current predictions, response rates are likely to decline further in the future [3]. While statistical methods, like weighting or imputation, are useful to substitute missing responses, it remains questionable whether they are applicable to studies in which respondents only represent a small fraction of the sample population. It is therefore crucial to explore methods that improve questionnaire response rates to population studies.

Much research has already been done to explore methods to improve response rates to postal questionnaires [5,6]. To date, the most effective strategies are known to be incentives, the use of special or certified delivery services or shorter questionnaires [5]. Pre-notification of receiving a questionnaire, reminder mailings and personalization methods can also increase questionnaire response rates significantly. However there are limitations to the existing literature on response rates. It is not clear to what extent methods that have proven to be effective for specific groups, for example, physicians or students are effective for general populations. Secondly, evidence collected previously for approaches based on newer technologies such as the internet may no longer be valid due to the wider use of those technologies within society. Finally the cost-effectiveness of different methods should also be assessed to allow researchers to choose the most appropriate overall approach.

A method that can easily be applied in population-based studies is to change the printing format of the questionnaire. As yet, four randomized controlled trials (RCTs) have examined the effect on response rates when questionnaires were printed single-sided versus double-sided [5]. Their findings suggest that the distribution of single-sided questionnaires leads to a small but significant effect on response rates (OR = 1.22; 95% CI: 1.01, 1.47). This may seem counter-intuitive since shorter questionnaires have shown to increase response rates. However, it may be speculated that questionnaire respondents felt more encouraged to complete the instrument after noticing that only the first side of the questionnaire pages was printed. They may have believed that its completion would not take up too much of their time after all, hence filled it in and returned it. None of the four trials were conducted within a general population sample. It would therefore be interesting to test this strategy in a population-based study.

The provision of an internet option to respond to a paper questionnaire may be another potential method to increase the response rate to population-based studies if prospective respondents prefer one administration mode over the other. The effect on response rates when participants received an option to reply via the internet was summarized in a recent meta-analysis [7]. Only four out of twelve peer-reviewed studies were RCTs that were conducted within general population samples [8-11]. The pooled Odds Ratio suggests that an additional web option does not improve response rates to population-based studies (OR = 0.90; 0.73, 1.11). Two out of the four trials were carried out more than five years ago. In the context of rapidly growing internet penetration rates (in Scotland: 62.7% in 2007 and 77.4% in 2012; [12]), it is possible that an additional response option could become more acceptable and in this way increase questionnaire response rates.

The aims of the current study were to explore the effectiveness of printing the questionnaires single-sided and providing an internet option to respond and to assess the cost-effectiveness of both methods in a population-based study. It was hypothesized that the distribution of single-sided questionnaires and the provision of an internet option have an independent effect on questionnaire response rates, while no interaction effect between methods was expected. A secondary aim was to use additional data collected in this study to update an earlier meta-analysis of the evidence on the effect of an internet option in population studies [7].

## Methods

### Study design

A 2×2 factorial experiment was embedded within a cross-sectional population-based study of pain and pain management in 2012/2013. Using an electronic randomization program, persons in the study sample were randomly allocated to either receive (1) a single-sided or double-sided questionnaire and (2) an option or no option to reply via the internet.

### Study procedure

**Figure 1:**
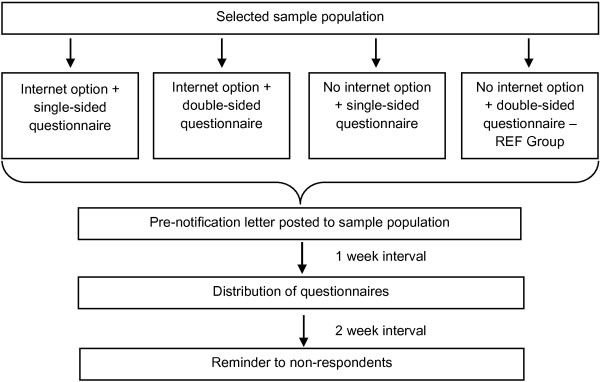
Flowchart of the study procedure.

A random sample of 4600 residents in Grampian (north of Scotland, UK) aged 25 years and over was selected from health board records. Upon randomization, selected persons were sent a notification letter that they had been selected for the study. One week later they were sent a survey pack comprising an invitation letter, an information sheet, the questionnaire and a pre-paid reply envelope. Potential participants were advised to read the information sheet and to complete and return the questionnaire if they wished to take part. The invitation letter for those in the web option groups contained, in addition, the URL link to the electronic questionnaire and their individual ID number and password for its access. Non-respondents were sent a second survey pack appropriate to their randomization group, three weeks after the first contact (i.e. two weeks after the questionnaire distribution) (Figure 1).

### Study questionnaire

The 20-page study questionnaire included questions on participants’ demographic characteristics (gender, age, and educational background), their health, pain and pain management. Validated standard questionnaires were used to collect information on participants’ health (SF-36 [13]) and pain status (set of pain questions with manikins and Chronic Pain Grade [14]). The questionnaire to collect pain management information was validated within a small population sample before the conduct of the study (Results to be published). Depending on their randomization, persons in the study sample either received a 10-sheet (double-sided) or a 20-sheet (single-sided) questionnaire.

### Statistical analysis

The main outcome of interest was questionnaire response rate; defined as the percentage of completed and partially completed questionnaires returned after excluding from the denominator those which were invalid (e.g. change of address or death). With questionnaire response as the dependent variable, logistic regression models were performed in order to determine predictors for questionnaire response. Odds Ratios and 95% Confidence Intervals were calculated in order to quantify the effect of the strategies on the questionnaire response rate. A meta-analysis was performed to summarize the current evidence on the effectiveness of an internet option in increasing response rates to population-based studies. Additionally, the cost-effectiveness of single-sided questionnaires and an internet option was investigated by determining the cost per additional response.

### Ethical approval

The study was approved by the North of Scotland National Health Service Research Ethics Committee (REC reference: 12/NS/0079).

## Results

### Response rate

The response rate before the reminder surveys were distributed to non-respondents was 21.4%. At the end of data collection, 183 questionnaires were found invalid (for example, due to an invited participant being deceased or no longer resident at the given address) and 1604 completed questionnaires were returned giving an overall study response rate of 36.3% (1604/4417). Out of the respondents who were offered an internet option, 60 completed the electronic questionnaire (7.3%).

### Time to respond

Completed study questionnaires were returned a median of 10 days (Interquartile range (IQR): 7, 20) after their distribution. Electronic questionnaires were received earlier than paper questionnaires (median, web: 7 days; median, paper: 10 days; Mann–Whitney test: p < 0.001).

### Respondents’ demographic characteristics

Females represented 55.3% of respondents. The median age was 55 years (IQR: 44, 65) with the oldest respondent aged 94 years. The majority of respondents reported secondary school (29.6%), a vocational qualification (20.0%) or a professional qualification (16.7%) as their highest level of education. When compared to non-respondents, respondents were significantly more likely to be female and of older age (Chi-square test: p < 0.001, both tests). There was a significant interaction between gender and age, showing that the positive effect of female gender on response reduces with older age. Electronic respondents (n = 60) were significantly more likely to be younger and more educated when compared to paper respondents (Chi-square test: p < 0.001, both tests).

### Response rate by study group

There were small differences in response rates between the four study groups. The reference group achieved a response rate of 35.5%. When sampled persons were provided with an internet option, this dropped to 34.3%. In contrast, the response rate increased to 35.8% when participants were sent a single-sided questionnaire with no option to respond via the internet. A greater increase in response rate of 4.1% was determined when participants received both interventions (39.6%).

### The effectiveness of the strategies in increasing response rates

From the logistic model there was no “main” effect on response rates when an internet option was provided (OR = 0.95, 95% CI = 0.80, 1.13) or when single-sided questionnaires were distributed (OR = 1.01, 95% CI = 0.85, 1.20). Moreover, the interaction between both strategies led to a larger, albeit not statistically significant, effect (OR = 1.24, 95% CI = 0.97, 1.59, Table 1).

**Table 1:** The effect of the strategies on response rates

**Logistic Regression, Model term**	**Crude OR (95% CI)**
No intervention	1.00
Web (main effect)	0.95 (0.80, 1.13)
Single-sided (main effect)	1.01 (0.85, 1.20)
Web + Single-sided (interaction term)	1.24 (0.97, 1.59)

When comparing the response rates of those who were randomized to receive an internet option and those who were not, the strategy of providing an internet option increased the response rate non-significantly by 1.3% (OR = 1.06; 95% CI: 0.94, 1.20). A somewhat larger, yet non-significant effect was determined when the response rates of those who were allocated to receive a single-sided questionnaire and those who were allocated to be sent a double-sided questionnaire were compared: the response rate increased by 2.8% when participants received a single-sided questionnaire (OR = 1.13; 95% CI: 0.998, 1.28).

### Internet option – summary of evidence

**Figure 2:**
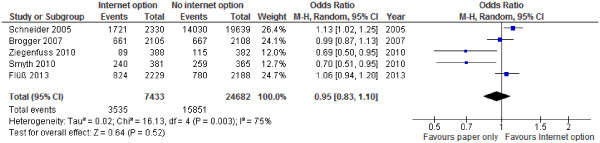
Comparison of an internet option vs. no internet option to increase response to population studies.

When the results of the current study were added to those reported in previous population studies (Figure 2), there no effect on response rates when an internet option versus no internet option was provided for participants (OR = 0.95; 0.83, 1.10). There was no obvious pattern of an improvement in the effectiveness of an internet option over time. There was significant heterogeneity between studies.

### Cost-effectiveness

The employment of the strategies to increase response rates was associated with additional cost. The use of single-sided questionnaires led to increased cost for printing (since the required number of pages doubled) and postage (because the survey packs were heavier). The implementation of an electronic version of the study questionnaire resulted in extra costs due to programming time and set up cost. Due to the low uptake rate of the internet option, differences in data entry costs were minimal. It was therefore decided not to include the data entry costs in the cost-effectiveness analysis. As outlined in Table 2, the cost per response was the lowest in the reference group that did not receive either intervention (£6.64). An additional cost of at least £1.02 per response was spent for the employment of the interventions. The internet option resulted in lower response at greater cost when double-sided questionnaires were distributed. The use of single-sided rather than double-sided questionnaires cost £119.19 per additional response. The combination of both methods was more cost-effective: Distributing single-sided questionnaire with an option to reply via the internet cost £25.20 per extra response.

**Table 2:** Strategies to improve questionnaire response: Cost-effectiveness

	**No internet option & Double-sided**	**Internet option & Double-sided**	**No internet option & Single-sided**	**Internet option & Single-sided**
**Response rate (in %)**	35.5	34.3	35.8	39.4
**Nr. of respondents**	393	382	387	442
**Nr. of eligible participants**	1106	1082	1113	1116
**Cost per response (£)**	6.64	8.59	7.67	8.36
**Cost per additional response (£)**	--	--	119.19	25.20

## Discussion

### Main findings

Overall, neither the use of single-sided questionnaires nor the provision to reply via the internet significantly improved the questionnaire response rate in the current study. A small but non-significant interaction effect was determined when both strategies were used in combination.

The current study was the first study that explored the effectiveness of single-sided questionnaires in a population-based sample. We found no effect on response rates when questionnaires were printed single-sided (OR = 1.13; 95% CI: 0.998, 1.28). Likewise, no effect on response rates was determined when an internet option was provided for the sampled persons (OR = 1.06; 95% CI: 0.94, 1.20). An updated meta-analysis confirmed that based on the current evidence of five studies, an option to reply via the internet does not improve response rates to a population study (OR = 0.95; 0.83, 1.10).

### Strengths and limitations

The current study had several strengths. Firstly, we used a randomized controlled study design. Secondly, the use of a two by two factorial design enabled us to test the independent and the combined effect of two different methods to increase questionnaire response rates. Thirdly, we conducted a subsequent cost-effectiveness analysis which provided insight into the expenses that were associated with the respective strategies under investigation.

A limitation of the study was that it was a sub-study within a large cross-sectional study. The study sample size was therefore powered on the main outcome variables and not on the difference in questionnaire response rates. Nevertheless, the main effects of both strategies on questionnaire response rates were very small and insufficient power to detect a meaningful difference is unlikely to have been a reason for observed lack of effect. It is probable however, that there was a lack of power to detect a significant interaction effect between both studies. Secondly, due to a small number of electronic responses the interpretation of some results was limited. It was determined that electronic respondents were significantly younger and more highly educated when compared to paper respondents. While a higher proportion of electronic respondents were male and living in an urban area (information provided by the sampling frame), these differences were not statistically significant – most probably due to a lack of power.

### Findings in relation to previous studies

Out of the 824 respondents who were randomized to the internet option groups, only 60 (7.3%) used the electronic version to respond to the questionnaire. The low uptake rate of the internet option was similar to that observed in the population study by Ziegenfuss et al. [9], in which only 8.0% of the web option respondents used the electronic questionnaire. While a lack of internet coverage was not a concern in the current study, it is likely that the initial mode of contact caused the low internet uptake rate. Since all prospective respondents were sent a paper questionnaire, they may have been more inclined to complete and return the paper version.

A couple of hypotheses have been discussed in the literature to explain why an internet option is ineffective in increasing response rates [7]. Firstly, when approached by mail, the completion of an electronic questionnaire requires additional effort for participants since they have to ‘switch task mode’. It can be hypothesized that a number of people may initially have decided to use the electronic questionnaire but never actually logged on to complete and submit. Secondly, long and complicated URLs (e.g. those containing numbers and case sensitive letters) may make it more difficult for participants to access the online questionnaire and appear off-putting. As a result, fewer participants may consider completing the online version. Nevertheless, the URL in the current study was reasonably short and only contained lowercase letters). Thirdly, people may be less likely to respond when provided with an internet option because they are required to make a choice at the very beginning. According to Schwartz [15], offering choices has negative effects on people’s decision-making. Since every choice is associated with opportunity costs, one must consider the trade-offs concerned with each option they are offered. This, in turn, makes it less appealing to choose either one. It remains uncertain why the use of single-sided questionnaires was ineffective in the current study. The questionnaire in the current study consisted of 20 pages and appeared very bulky when printed single-sided. It can be assumed that people might have felt discouraged when they received the questionnaire. Surprisingly, we established that although not statistically significant, there was an effect on questionnaire response rate that laid in the interaction of both strategies (OR = 1.24, 95% CI = 0.97, 1.59). It remains questionable why the response rates were only increased when sampled persons received both interventions. It can be speculated that fewer people considered using the internet option when they were sent a single-sided questionnaire. People may have believed that its completion would take up less of their time using the printed version and hence, were more likely to complete and return it straight away.

We added our findings to the results of previous population studies that were summarized in a recent meta-analysis by Medway and Fulton [7]. In order to account for any studies that were published after 2011, we conducted a comprehensive literature search. However, no additional papers were identified.

## Conclusions

Taken as a whole, neither an option to reply via the internet nor the use of single-sided questionnaires were effective methods to significantly increase the response rates to a population-based sample. The results derived from the web option comparison were in agreement with the pooled effect size of the current evidence. Furthermore, it has been demonstrated that the cost per additional response was high, even when both methods were combined. As result of the current findings, researchers should be aware that neither method is effective in increasing response rates to future population studies. Besides, researchers should be alerted to select the methods to increase questionnaire response rates in consideration of their effectiveness and the costs concerned. Due to the current trend of decreasing response rates, further research into strategies to increase those remains important. Future studies should always provide sufficient information on the cost-effectiveness of each employed strategy in order to allow an appropriate assessment of these.

## Competing interests

The authors declare that they have no competing interests.

## Authors’ contributions

This study was conceived by GJM, GTJ and CMB. It was planned by all the authors and EF conducted the study. EF drafted the manuscript and all other authors critically reviewed the manuscript and provided important intellectual content. All authors read and approved the final manuscript.

## Pre-publication history

The pre-publication history for this paper can be accessed here:

http://www.biomedcentral.com/1471-2288/14/104/prepub
